# 中国多发性骨髓瘤患者治疗相关生活质量量表的编制与信效度检验

**DOI:** 10.3760/cma.j.cn121090-20241017-00399

**Published:** 2025-08

**Authors:** 春艳 孙, 真 蔡, 兵 陈, 丽娟 陈, 文明 陈, 凯阳 丁, 鹃 杜, 蓉 付, 琤琤 傅, 大 高, 广勋 高, 艳娟 贺, 健 侯, 明 江, 菲 李, 剑 李, 娟 李, 振宇 李, 爱军 廖, 竞 刘, 军 罗, 建民 罗, 艳萍 马, 坚青 糜, 挺 牛, 宏凌 彭, 永平 宋, 鲁群 王, 榕 战, 曦 张, 豫 胡

**Affiliations:** 1 华中科技大学同济医学院附属协和医院，武汉 430022 Union Hospital, Tongji Medical College, Huazhong University of Science and Technology, Wuhan 430022, China; 2 浙江大学医学院附属第一医院，杭州 310003 The First Affiliated Hospital of Zhejiang University, Hangzhou 310003, China; 3 南京大学医学院附属鼓楼医院，南京 210008 Nanjing Drum Tower Hospital, Nanjing University Medical School, Nanjing 210008, China; 4 江苏省人民医院，南京 210029 Jiangsu Provincial Hospital, Nanjing 210029, China; 5 首都医科大学附属北京朝阳医院，北京 100021 Beijing Chaoyang Hospital, Capital Medical University, Beijing 100021, China; 6 安徽省立医院，合肥 230001 Anhui Provincial Hospital, Hefei 230001, China; 7 上海长征医院，上海 200240 Shanghai Changzheng Hospital, Shanghai 200240, China; 8 天津医科大学总医院，天津 300052 Tianjin Medical University General Hospital, Tianjin 300052, China; 9 苏州大学附属第一医院，苏州 215006 The First Affiliated Hospital of Soochow University, Suzhou 215006, China; 10 内蒙古医科大学附属医院，呼和浩特 010059 Affiliated Hospital of Inner Mongolia Medical University, Hohhot 010059, China; 11 空军军医大学西京医院，西安 710032 Xijing Hospital, Air Force Medical University, Xi'an 710032, China; 12 中南大学湘雅医院，长沙 410008 Xiangya Hospital, Central South University, Changsha 410008, China; 13 上海交通大学医学院附属仁济医院，上海 200127 Renji Hospital, School of Medicine, Shanghai Jiaotong University, Shanghai 200127, China; 14 新疆医科大学第一附属医院，乌鲁木齐 830054 The First Affiliated Hospital of Xinjiang Medical University, Urumqi 830054, China; 15 南昌大学第一附属医院，南昌 330006 The First Affiliated Hospital of Nanchang University, Nanchang 330006, China; 16 中国医学科学院北京协和医院，北京 100730 Peking Union Medical College Hospital, Chinese Academy of Medical Sciences, Beijing 100730, China; 17 中山大学附属第一医院，广州 510080 The First Affiliated Hospital of Sun Yat-sen University, Guangzhou 510080, China; 18 徐州医科大学附属医院，徐州 221002 Affiliated Hospital of Xuzhou Medical University, Xuzhou 221002, China; 19 中国医科大学附属盛京医院，沈阳 110004 Shengjing Hospital of China Medical University, Shenyang 110004, China; 20 中南大学湘雅三医院，长沙 410013 Third Xiangya Hospital, Central South University, Changsha 410013, China; 21 广西医科大学第一附属医院，南宁 530021 The First Affiliated Hospital of Guangxi Medical University, Nanning 530021, China; 22 河北医科大学第二医院，石家庄 050011 The Second Hospital of Hebei Medical University, Shijiazhuang 050011, China; 23 山西医科大学第二医院，太原 030001 The Second Hospital of Shanxi Medical University, Taiyuan 030001, China; 24 上海交通大学医学院附属瑞金医院，上海 200025 Ruijin Hospital Affiliated to Shanghai Jiaotong University School of Medicine, Shanghai 200025, China; 25 四川大学华西医院，成都 610041 West China Hospital, Sichuan University, Chengdu 610041, China; 26 中南大学湘雅二医院，长沙 410011 Second Xiangya Hospital, Central South University, Changsha 410011, China; 27 郑州大学第一附属医院，郑州 450052 The First Affiliated Hospital of Zhengzhou University, Zhengzhou 450052, China; 28 山东大学齐鲁医院，济南 250012 Qilu Hospital of Shandong University, Jinan 250012, China; 29 福建医科大学附属协和医院，福州 350001 Union Hospital of Fujian Medical University, Fuzhou 350001, China; 30 陆军军医大学新桥医院，重庆 400010 Chongqing Xinqiao Hospital, Chongqing 400010, China

**Keywords:** 多发性骨髓瘤, 生活质量, 量表, 结果可重复性, Multiple myeloma, Quality of life, Scale, Reproducibility of results

## Abstract

**目的:**

编制适用于中国多发性骨髓瘤（MM）患者的治疗相关生活质量量表，并检验其信效度。

**方法:**

通过文献检索、德尔菲专家函询及认知测试构建初始版量表。2024年2月至2024年3月在全国共155个医院的血液内科选取379例MM患者进行预调查，选取865例患者进行正式调查，对量表进行条目分析及信效度检验，形成最终版量表。

**结果:**

中国MM患者治疗相关生活质量量表包含生理、心理、社会、治疗不良反应、总体健康和其他6个维度，共36个条目。预调查阶段，量表各条目的Cronbach's *α*系数为0.597～0.939，量表重测信度为0.747（*P*<0.001）。探索性因子分析提取8个公因子，累计方差贡献率为60.058％。正式调查阶段，量表各条目的Cronbach's *α*系数为0.484～0.930，量表重测信度为0.835（*P*<0.001）。验证性因子分析结果显示，比较拟合指数为0.750，近似误差均方根为0.090，均方根残差为0.067。

**结论:**

中国MM患者治疗相关生活质量量表能反映治疗对患者生活质量的影响，具有良好的信效度，可为临床医师评估患者的疾病状况提供参考。

## Abstract (en)

**Objective:**

To develop a treatment-related quality of life scale for Chinese patients with multiple myeloma（MM）and to test its reliability and validity.

**Methods:**

The initial scale was constructed through a literature search, Delphi expert correspondence, and cognitive testing. This study conducted a preliminary survey of 379 patients with MM and a formal survey of 865 patients from the hematology departments of 155 hospitals nationwide from February 2024 to March 2024. The final scale was obtained after conducting item analysis and reliability and validity tests on the initial scale.

**Results:**

The constructed scale contains 36 items covering six domains: physiological, psychological, social, treatment side effects, general health, and others. In the preliminary survey, the Cronbach's alpha coefficient of each item ranged from 0.597 to 0.939, and the test-retest reliability was 0.747（*P*<0.001）. Exploratory factor analysis extracted eight common factors with a cumulative variance contribution of 60.058%. In the formal survey, the Cronbach's alpha coefficient of each item ranged from 0.484 to 0.930, and the test-retest reliability was 0.835（*P*<0.001）. Confirmatory factor analysis revealed a comparative fit index of 0.750, a root-mean-square error of approximation of 0.090, and a root-mean-square residual of 0.067.

**Conclusion:**

The treatment-related quality of life scale for Chinese patients with MM designed in this study exhibited good reliability and validity, reflecting the impact of treatment on the quality of life of patients. This scale can provide a reference to clinicians for assessing the disease status of patients.

多发性骨髓瘤（multiple myeloma, MM）是一种克隆浆细胞异常增殖的血液系统恶性肿瘤，常见症状为贫血、溶骨性病变、肾损伤和高钙血症等[1]–[2]。全球MM疾病负担研究的分析结果显示，MM发病率为（3～6）/100 000，5年患病率为（7～14）/100 000[3]。我国MM患者的发病率约为1.15/100 000，2022年死亡病例约1.7万[4]–[5]。随着新药物和新技术的不断涌现，MM的临床治疗进入高速发展时期，患者总生存期已延长至7～10年[6]–[7]。在生存期延长的同时，MM患者可能出现更多的症状和不良反应，影响患者的生活质量，已成为MM患者治疗中日益突出的问题[8]。因此，患者报告结局（patient reported outcomes，PRO）作为获取患者生活质量情况的重要方式，在MM患者中逐渐受到重视。

目前已有多种PRO量表用于MM患者的生活质量评估[9]–[11]，但仍缺乏指导中国MM患者治疗的PRO量表。如欧洲肿瘤治疗研究中心（EORTC）研发的MM患者生活质量评价量表（EORTC QLQ-MY20）中，症状和治疗不良反应方面的评估以骨骼关节疼痛、手足刺痛和感觉不适为主[9]，而社会支持和预后方面的评估因社会人文因素并不适用于中国患者，且目前并没有中文版EORTC QLQ-MY20量表的信效度验证研究。此外，EORTC研发的肿瘤患者生命质量测定量表（EORTC QLQ⁃C30）在MM患者中的使用频率也较高，包括认知、情感、疼痛、疲劳、呼吸困难等评估条目，但EORTC QLQ-C30适用于多种癌症评估而非MM患者专用量表。因此，亟需开发适用于我国MM患者的PRO报告工具。本研究旨在编制和验证中国MM患者治疗相关生活质量量表，准确有效地评估中国MM患者的治疗效益，降低其面临的治疗负担，提高患者的整体生活质量。

## 病例与方法

一、中国MM患者生活质量量表的编制

考虑到MM好发于老年人，故量表条目数不宜过多，且需要考虑患者的合并症及疾病的特异性。本研究通过以下三个步骤进行量表的编制：①条目池构建：通过文献查阅、筛选及条目提取构建量表条目池；②专家函询：通过两轮德尔菲专家函询筛选条目；③认知测试：评估量表条目可理解性、准确性及总体情况。

本研究已获得华中科技大学同济医学院附属协和医院伦理委员会的批准［批准文号：（2023）伦审字（0995）号］，参与问卷填写的患者均提供了知情同意书。

1. 条目池构建：以“多发性骨髓瘤”、“患者报告结局/生活质量/生命质量”、“维度/量表/问卷/调查”、“生理/心理/社会/副作用/症状/功能”为中文检索词，以“multiple myeloma/myelomatosis”、“patient reported outcomes/quality of life/life quality”、“scope/score/scale/item/measure/questionnaire”、“physiological/psychosocial/social/side-effect/functional/symptom”为英文检索词，制定检索策略。从四个中英文数据库（PubMed、Embase、中国知网和万方数据库）检索既往已经发表的文献，检索时限为2018年1月1日至2023年7月21日。经去重和筛选，在纳入的文献中汇总既往报道的MM患者相关PRO量表，提取频次较高的量表条目构建条目池，合并同类条目后，条目池总计183个条目。

本次量表的编制依据世界卫生组织对健康的定义及医学结局研究（MOS）框架[12]，经过MM专家讨论，确定中国MM患者生活质量评估量表（PRO-MM）。包括以下六个维度：①生理维度；②心理维度；③社会维度；④治疗不良反应维度；⑤总体健康维度；⑥其他维度。PRO-MM量表参考EORTC QLQ⁃C30设置4个选项：没有、有一点、比较多、非常多。在评分时正向条目按1～4分计分，没有：1分；有一点：2分；比较多：3分；非常多：4分。逆向条目则反向计分，即选择第1个选项计4分，选择第2个选项计3分，以此类推。将各维度条目得分相加，除以回答的条目数，即可得到该维度的得分。各维度得分相加得到量表总分。

2. 专家函询：通过德尔菲专家函询，征询专家关于PRO量表的意见，对量表维度和条目进行增加、删减及语言调适。本研究共进行了两轮德尔菲专家函询。专家来自全国29个医院的血液内科。专家遴选标准：①从事MM诊疗工作≥5年；②本科及以上学历；③中级及以上职称；④自愿参加本项目。量表条目池共计183个条目，第一轮函询后，有71个条目因不符合筛选标准［均值<4分，满分比<40％，变异系数（CV）>30％］被删除，共初筛出112个条目。第二轮函询后，有53个条目因不符合筛选标准（均值<4分，满分比<35％，CV>30％）被删除，再结合专家咨询意见，增加2个条目（您的血压是否受到影响、您是否有皮疹），故第二轮函询结束后共有61个条目进入认知测试。

3. 认知测试：本研究共选取30例MM患者进行认知测试，收集患者对量表问题、选项的理解程度和准确性评分及对量表的总体评价，同时考量量表的作答时长及应答率。根据认知测试结果进一步调整量表维度和条目并进行语言调适。

二、中国MM患者生活质量量表的信效度检验

首先使用初始测试版PRO-MM量表对MM患者进行小样本预调查，采用探索性因子分析（exploratory factor analysis, EFA）筛选条目和初步评价量表的信度、效度；再使用正式测试版PRO-MM量表对MM患者进行大样本正式调查，采用验证性因子分析进一步评价量表的信度、效度和可行性。

1. 调查对象：根据相应纳入和排除标准，2024年2月至2024年3月在全国共155个医院的血液内科招募MM患者进行预调查和正式调查。纳入标准包括（符合以下所有条件）：①年龄≥18岁，性别不限；②根据国际骨髓瘤工作组（IMWG）2016年更新的标准确诊为MM[13]，且目前正在接受相关治疗；③自愿参与并签署知情同意书。排除标准包括（符合以下任意1条）：①罹患除MM以外的恶性肿瘤；②患有精神疾病或严重情绪障碍，可能影响量表正确评分；③患有认知功能障碍；④存在严重心、脑、肺、肝、肾功能衰竭；⑤研究者认为不能配合完成量表或难以沟通。

根据样本量计算方法[14]，样本量应为量表条目数的5～10倍，同时考虑20％的无效应答率，本研究初始版量表共52个条目，故计算样本量为325～650例。

2. 问卷发放和数据收集：由医师邀请MM患者填写预调查问卷和正式调查问卷。收集招募患者的社会人口学资料（包括性别、年龄、婚姻状况、学历和就业状况），疾病相关资料（疾病目前所处阶段、治疗方案），并填写PRO-MM量表和效标量表EORTC QLQ-C30。

3. 条目分析方法：采用离散趋势法、条目分布考察法、条目维度相关系数法、Cronbach's *α*系数法、因子分析法对条目进行分析，进一步筛选量表条目。其中，Cronbach's *α*系数法为信度检验方法，因子分析法为效度检验方法。其他3种分析方法的删除标准如下：①离散趋势法：删除CV<0.3的条目[15]。②条目分布考察法：删除答案呈明显偏态分布的条目（即该条目答案的受试者选择率大于80％）[15]。③条目维度相关系数法：删除相关系数<0.4的条目[16]。

4. 信度检验方法：①通过计算总量表及各维度的Cronbach's *α*系数，考察量表条目之间的一致性信度，将Cronbach's *α*系数<0.7的条目删除[15]。②重测信度采用组内相关系数（intraclass correlation coefficient，ICC）进行评价，ICC>0.7表示量表重测信度良好[16]。在预调查阶段，从首次参与调查的MM患者中随机选取50例间隔2周后再次填写量表，以检验量表的重测信度。自首次填写到再次填写量表期间，患者接受的治疗方案保持稳定不变。正式调查阶段同样进行了重测信度检验。

5. 效度检验方法：

（1）内容效度：本量表的研制方法依据我国国家药监局药审中心发布的PRO测量量表的应用指导原则。在查阅大量国内外文献及咨询专家的基础上构建了量表的理论框架。通过专家咨询法和患者认知测试法对条目进一步筛选和优化，获得量表评价的最终指标，能够反映我国MM患者的真实情况。经过专家研讨会反复讨论，对量表语言表达的准确性进行调试。在此基础上对量表的结构、内容和临床可操作性进行评估，最终形成具有较好代表性的量表。

（2）结构效度：预调查阶段采用EFA检验量表的结构效度。EFA考虑删除条目的标准是：①条目在每个因子上载荷均<0.5；②对其归属因子以外的其他因子载荷>0.5[14]–[15]。

正式调查阶段采用验证性因子分析检验量表的结构效度，主要探讨因子载荷，并通过检查模型*χ*^2^/*df*、比较拟合指数（comparative fit index，CFI）、近似误差均方根（root-mean-square-error of approximation，RMSEA）和均方根残差（root-mean-squared residual，RMR）评估整体模型的拟合程度。这些指数的理想拟合标准包括：*χ*^2^/*df*为1～3表示适配良好、CFI>0.8、RMR<0.08和RMSEA<0.08[17]。

（3）效标关联效度：以EORTC QLQ-C30得分为效标，采用Spearman相关分析检验PRO-MM量表总分、各维度得分与EORTC QLQ-C30总分的相关性，相关系数越高，表明效标关联效度越好[14]–[15]。

6. 统计学处理：统计软件为IBM SPSS 22.0，定量资料采用*x*±*s*和*M*（*Q*_1_，*Q*_3_）表示，分类资料用频数（％）表示。计量资料的正态性检验采用Shapiro-Wilk方法。使用Spearman秩相关方法计算两组非正态计数资料间的相关系数。所有检验均为双侧检验，检验水准*α*＝0.05，*P*<0.05为差异有统计学意义。

## 结果

一、专家函询及认知测试结果

第一轮函询共有13名专家参与，专家的中位年龄为50（30，63）岁，中位工作年限为17（5，36）年。第二轮函询共有18名专家完成问卷调查，15名专家的年龄>50岁，18名专家的中位工作年限为19（10，35）年。经德尔菲专家函询可知，专家意见的权威性、协调性和一致性均较好，说明本研究方法可行，本次研究结果可信。

认知测试中患者理解程度中等或偏低的条目共25个，经专家讨论后删除9个条目，最终形成包含52个条目的初始测试版量表。此外，经专家讨论，将条目选项“有一些”改为“比较多”，并修改3个条目。

二、预调查结果

1. 调查对象的一般资料：在预调查阶段，共收集379份MM患者的有效问卷。参与预调查的患者中男性患者209例（55.1％），中位年龄为63（57，70）岁。326例（86.0％）患者已婚，本科及以上学历65例（17.1％）。127例（33.5％）患者目前正处于诱导治疗后的维持治疗期，接受蛋白酶体抑制剂治疗的患者245例（64.6％）（表1）。

**表1:** 参与预调查和正式调查的多发性骨髓瘤患者的一般人口学资料

指标	预调查 （379例）	正式调查 （865例）
年龄［*M*（*Q*_1_，*Q*_3_）］	63（57，70）	64（56，70）
性别［例（％）］		
男	209（55.1）	484（56.0）
女	170（44.9）	381（44.0）
婚姻状况［例（％）］		
已婚，有配偶	326（86.0）	731（84.5）
丧偶	32（8.4）	79（9.1）
离异	11（2.9）	30（3.5）
未婚	10（2.6）	25（2.9）
最高学历［例（％）］		
小学	102（26.9）	253（29.2）
初中	124（32.7）	258（29.8）
高中	88（23.2）	183（21.2）
大学	58（15.3）	145（16.8）
研究生及以上	7（1.8）	26（3.0）
就业状况［例（％）］		
退休	202（53.3）	397（45.9）
无业	124（32.7）	317（36.6）
在职	49（12.9）	148（17.1）
在读	4（1.1）	3（0.3）
所处疾病阶段［例（％）］		
新诊断（刚确诊）	50（13.2）	102（11.8）
诱导治疗期	53（14.0）	167（19.3）
不适合移植患者的维持治疗期	127（33.5）	246（28.4）
造血干细胞移植后维持治疗期	42（11.1）	116（13.4）
造血干细胞移植后巩固治疗期	19（5.0）	29（3.4）
首次复发/原发耐药	41（10.8）	97（11.2）
多线复发	38（10.0）	86（9.9）
仅支持治疗^a^	9（2.4）	22（2.5）
接受的治疗类型［例（％）］		
蛋白酶体抑制剂	245（64.6）	534（61.7）
免疫调节剂	153（40.4）	385（44.5）
支持治疗	97（25.6）	233（26.9）
生物制剂	24（6.3）	111（12.8）
细胞毒性药物	13（3.4）	50（5.8）
其他抗肿瘤药物	44（11.6）	105（12.1）

**注** ^a^包括输注红细胞、纠正贫血、纠正高钙血症、预防感染等

预调查阶段量表共52个条目，中位答题时间为7.00（4.17，11.79）min，总体可行。量表中所有条目答案的选择率均≤80％，说明答案未呈现明显偏态分布，故所有条目均纳入后续分析。

2. 条目分析结果：在预调查阶段，采用离散趋势法、条目分布考察法、条目维度相关系数法、Cronbach's *α*系数法、探索因子分析法等5种方法对条目进行分析，以筛选量表条目。保留至少满足3种筛选方法的条目，3个条目不满足此条件被删除。另有12个条目因离散趋势较差、临床意义较小或与其他条目内容相似而被删除。预调查后量表共保留37个条目。

3. 信度检验结果：关于各维度的Cronbach's *α*系数，除了社会维度（*α*＝0.656）和其他维度（*α*＝−0.292），剩余4个维度的Cronbach's *α*系数均≥0.7（表2）。量表中每个条目的Cronbach's *α*系数为0.597～0.939。

**表2:** 预调查和正式调查阶段量表各维度总体Cronbach's *α*系数

维度	预调查	正式调查
生理维度	0.937	0.921
心理维度	0.702	0.634
社会维度	0.656	0.518
治疗不良反应维度	0.857	0.865
总体健康维度	0.822	–
其他维度	−0.292	−0.401

**注** –：无数据

关于重测信度，预调查共收集47例样本，经过统计分析，总体ICC为0.747（*P*<0.001），说明本量表重测信度较好。

4. 效度检验结果：预调查中KMO＝0.937、Bartlett's球形检验近似*χ*^2^值为11 490.025（*df*＝1326，*P*<0.001），均提示适合进行EFA。提取前8个因子，累积方差贡献率为60.058％（>60％），各因子特征值均>1。删除10个条目。

PRO-MM量表不同维度总分与EORTC QLQ-C30量表整体分数相关性分析结果显示，PRO-MM量表各维度与EORTC QLQ-C30量表的相关系数为0.383～0.813（*P*值均<0.01），说明本量表与效标量表关联效度较好。

三、正式调查结果

1. 调查对象的一般资料：参与正式调查的患者共865例，回收问卷865份，有效问卷865份，有效率为100％。正式调查阶段男性患者484例（56.0％），中位年龄为64（56，70）岁。已婚患者731例（84.5％），本科及以上学历171例（19.8％）。从疾病阶段和目前正在接受的药物治疗方案来看，246例（28.4％）患者目前正处于诱导治疗后的维持治疗期，534例（61.7％）患者正在接受蛋白酶体抑制剂治疗（表1）。

正式调查阶段量表共37个条目，中位答题时间为5.27（3.32，9.62）min，可行性较高。量表中所有答案的选择率均≤80％，说明答案未呈现明显偏态分布，故所有条目均纳入后续分析。

2. 条目分析结果：在正式调查阶段，同样采用离散趋势法、条目分布考察法、条目维度相关系数法、Cronbach's *α*系数法、验证因子分析法等5种方法对条目进行分析，进一步筛选量表条目。保留至少满足3种筛选方法的条目，有1个条目不满足此条件被删除。正式调查后量表共保留36个条目（表3）。

**表3:** 中国多发性骨髓瘤患者治疗相关生活质量量表 请回忆您过去4周是否有以下情况，并根据自身真实感受回答所有问题，并在最适合的选项上划√

问题	选项			
生理维度	没有	有一点	比较多	非常多
1. 当您做一些费力的动作，如提沉重的购物袋或行李箱时，您是否感到困难？				
2. 您是否能自己洗澡或穿衣？				
3. 长距离步行（1 000 m以上）时，您是否感到困难？				
4. 在白天，您是否必须卧床或坐在椅子上？				
5. 您是否有睡眠问题？				
6. 疼痛是否影响了您的日常生活或工作？				
7. 您的疼痛是否在活动后加重？				
8. 您的饮食有变化吗（没有胃口或过量进食）？				
9. 您曾感受到恶心想吐吗？				
10. 您是否感觉容易衰弱和疲乏？				
11. 您是否感到体重减轻？				
12. 您的手指或手部有无麻木感？				
13. 您的脚趾或者脚是否有针扎或者烧灼样疼痛？				
14. 您有无脚无力导致从椅子上站起来或爬楼梯有困难？				
15. 您是否因为生理健康原因，减少了工作量或活动量？				
16. 您是否经常感冒/感染？				
心理维度	没有	有一点	比较多	非常多
17. 您担心可能会出现新的疾病症状吗？				
18. 您很担心自己能否继续工作（包括在家工作）吗？				
19. 您是否感到情绪低落、沮丧，快乐不起来？				
20. 您是否对自己处理疾病的方式比较满意？				
21. 您是否觉得生活仍然是充满希望的？				
社会维度	没有	有一点	比较多	非常多
22. 您的业余爱好和休闲活动是否受到体能限制？				
23. 您的身体状况或治疗过程，妨碍了您的家庭生活吗？				
24. 您的家人能够接受您患病的事实吗？				
25. 您是否有工作能力？				
治疗不良反应维度	没有	有一点	比较多	非常多
26. 您是否感到昏昏欲睡？				
27. 您的脚趾或脚部有无刺痛感？				
28.您是否感到坐立不安或焦躁不安？				
29. 您是否有眼睛视力下降或视物模糊？				
30. 您是否有腹泻或便秘？				
31. 治疗的不良反应是否困扰着您？				
32. 您的血压是否受到影响？				
33. 您是否有皮疹？				
总体健康维度	很差	比较差	一般	很好
34. 与1年前相比，您如何评价现在的健康状况？				
其他维度	没有	有一点	比较多	非常多
35. 您的身体状况或治疗过程，造成了您的经济困难吗？				
36. 考虑药物疗效、费用和治疗副作用等，您对药物治疗是否满意？				

**注** 条目2、20、21、24、25、34、36为正向条目，其余均为逆向条目

3. 信度检验结果：生理维度的Cronbach's *α*系数为0.921，治疗不良反应维度的Cronbach's *α*系数为0.865，说明这两个维度内部一致性较好。其他维度只有2个条目，Cronbach's *α*系数为−0.401；总体健康维度仅1个条目，故无法计算Cronbach's *α*系数；心理维度和社会维度的Cronbach's *α*系数分别为0.634、0.518（表2）。量表中各条目的Cronbach's *α*系数为0.484～0.930。

关于重测信度，正式调查共收集51例样本，经过统计分析，总体ICC为0.835（*P*<0.001），再次验证了本量表重测信度较好。

4. 效度检验结果：根据正式调查验证性因子分析结果，CFI为0.750，RMSEA为0.090，RMR为0.067，表明本量表的结构效度尚可。验证性因子载荷结构模型见图1。

**图1:**
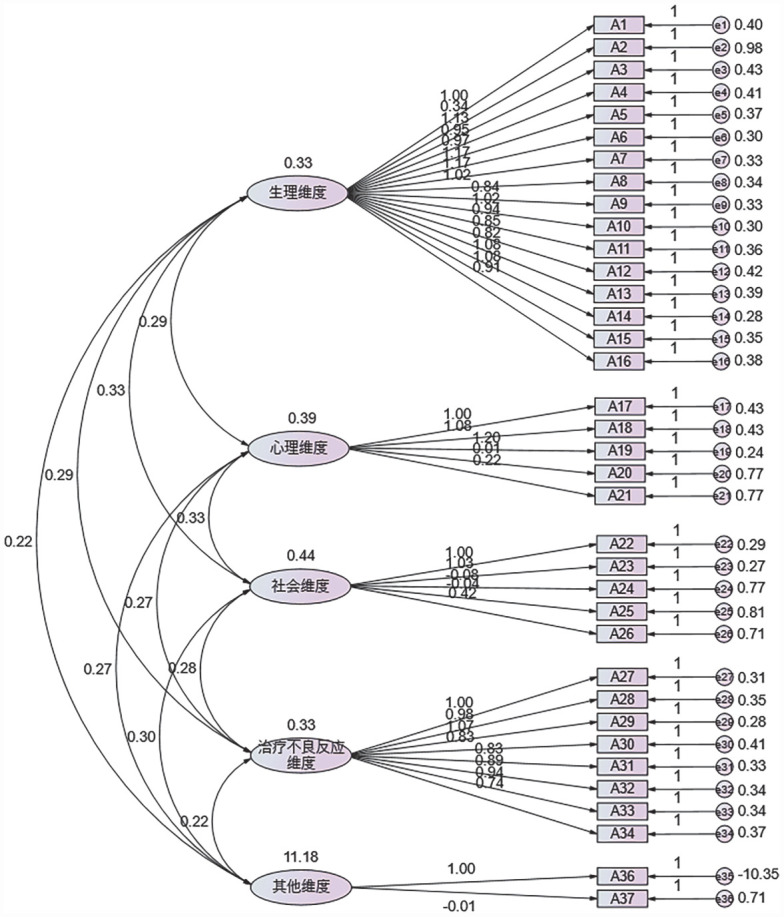
多发性骨髓瘤患者生活质量量表正式调查阶段验证性因子载荷结构模型

正式调查阶段PRO-MM量表与EORTC QLQ-C30量表各维度的相关系数为0.353～0.848（*P*值均<0.01），说明本量表与效标量表的关联效度仍然较好。

从整个编制过程来看，本量表研究方法规范，内容设置合理，以专家讨论结果和患者调查结果作为量表修改依据，因此本量表的内容效度较高。另外，结构效度可以间接反映内容效度，即结构效度尚可说明内容效度尚可。

## 讨论

本次量表编制之前曾开展一项针对中国MM患者和医师的调研。调研结果显示，PRO量表目前临床应用不广泛的主要原因是重视程度不够及医护人员对量表认知不足。医师认为PRO量表评估内容应集中于患者身体、心理和社会角色的功能状态，患者疾病症状报告及患者健康行为等方面。大多数MM患者表示未接受过PRO量表评估，但实际参与意愿强烈，然而目前国内并没有专门针对MM的中文版量表。基于此，本研究全面检索国内外文献，收集MM患者生活质量量表，提取条目构建条目池。采用德尔菲法对MM领域共31名专家进行2轮专家函询，结合专家咨询意见，初步完成MM患者治疗相关生活质量量表的编制。专家的遴选是保证德尔菲函询结果可靠性的关键。本研究中函询专家从事MM治疗相关工作的平均年限>15年，临床经验丰富，保证了函询结果的权威性。此外，函询专家还评估了量表条目的重要程度，并提出增加、删减、修改条目等意见，增加了函询结果的科学性。由此可见，PRO-MM量表的编制过程遵循了严格的标准，保证了量表的严谨性和科学性，使量表中各条目和维度具备合理性、专业性和代表性等。

近年来，新型治疗模式的出现使MM逐渐转变为慢性疾病，治疗目标也更关注改善或维持生活质量，实现患者高质量的长期无病生存[18]。患者在MM治疗过程中可能发生一系列不良反应，如周围神经病变、恶心、呕吐、腹泻等[19]。一项关于中国MM患者的横断面调查结果显示，EORTC QLQ-MY20量表未能体现多种治疗方案的不良反应对生活质量的影响，不足以指导治疗方案的选择[20]。国外研究者开发了多种MM患者生活质量评估量表，如癌症治疗功能评估（the Functional Assessment of Cancer Therapy，FACT）-MM量表，M.D. Anderson症状-MM（the M.D. Anderson Symptom Inventory-Multiple Myeloma，MDASI-MM）量表等，对治疗满意度的关注均较少[21]。此外，有研究表明EORTC QLQ-MY20量表可能会遗漏对患者很重要的社会心理问题[22]，且该量表不能计算总分，导致无法准确评估患者的总体情况。本量表在开发过程中系统收集国内外文献作为参考，再结合我国MM患者的具体情况进行了调整，增加了治疗不良反应（如高血压、皮疹等）条目，还增加了心理功能条目如对疾病复发的担忧、对生活的期望等。最终形成的PRO-MM量表共包含6个维度、36个条目，分别为生理维度（16个条目）、心理维度（5个条目）、社会维度（4个条目）、治疗不良反应维度（8个条目）、总体健康维度（1个条目）和其他维度（2个条目）。重点关注患者因疾病导致的躯体疼痛、恶心、失眠等生理问题，因治疗不良反应产生的情绪低落、焦虑等心理问题，因身体状况和治疗过程导致的社交活动受限等，以及总体健康评价和治疗满意度。

此外，PRO量表收集信息与研究设计的一致性、对统计方法描述的准确性及阈值设置的合理性对于促进PRO量表的临床应用至关重要[23]。值得注意的是，并非所有PRO量表均对血液系统恶性肿瘤患者进行信效度验证[24]。本量表在开发过程中进行了信度和效度检验，首先将379例MM患者作为预调查对象，采用5种分析方法对PRO-MM量表进行初步验证。条目“您要哭或想哭吗”、“您是否为便秘而感到烦恼”、“您是否因为头痛、颈痛和背痛而苦恼”因不满足筛选标准而被删除。量表中各条目的Cronbach's *α*系数为0.597～0.939，提示各条目的内部一致性较好。预调查结束后，将865例MM患者作为正式调查对象，同样采用5种分析方法进一步验证量表的信度、效度和可行性。条目“您在感情上得到家人的支持了吗”因不满足筛选标准而被删除。正式调查结果显示，量表的结构效度尚可，各条目之间存在显著相关性，拟合优度等也达到了统计学要求。重测信度和关联效度结果也表明本量表在整体上呈现较好的稳定性和可靠性。

一项针对MM患者的定性访谈结果显示，患者认为疾病状态、治疗因素、情绪状态、社会支持、未来期望等对生活质量有重要影响[25]。PRO-MM量表经过2轮人群验证，具有较好的信度和效度，可更加全面、准确地评估我国MM患者的生活质量。关于PRO-MM量表的实用性，该量表共36个条目，经过测试，作答时间为5～7 min，且语言简洁明了、易于理解，便于量表的临床应用。在未来，将扩大样本量以进一步验证PRO-MM量表的信度和效度，也期待能够将更加成熟的量表体系纳入国际科学共同体框架。
